# Lipid-Laden Macrophages in Pulmonary Diseases

**DOI:** 10.3390/cells13110889

**Published:** 2024-05-22

**Authors:** Yin Zhu, Dooyoung Choi, Payaningal R. Somanath, Duo Zhang

**Affiliations:** 1 Clinical and Experimental Therapeutics, College of Pharmacy, University of Georgia, Augusta, GA 30912, USAdooyoung.choi@uga.edu (D.C.);; 2 Charlie Norwood VA Medical Center, Augusta, GA 30912, USA; 3 Department of Medicine, Medical College of Georgia, Augusta University, Augusta, GA 30912, USA

**Keywords:** lipid-laden macrophage, pulmonary fibrosis, COPD, tuberculosis, lipid accumulation, reactive oxygen species

## Abstract

Pulmonary surfactants play a crucial role in managing lung lipid metabolism, and dysregulation of this process is evident in various lung diseases. Alternations in lipid metabolism lead to pulmonary surfactant damage, resulting in hyperlipidemia in response to lung injury. Lung macrophages are responsible for recycling damaged lipid droplets to maintain lipid homeostasis. The inflammatory response triggered by external stimuli such as cigarette smoke, bleomycin, and bacteria can interfere with this process, resulting in the formation of lipid-laden macrophages (LLMs), also known as foamy macrophages. Recent studies have highlighted the potential significance of LLM formation in a range of pulmonary diseases. Furthermore, growing evidence suggests that LLMs are present in patients suffering from various pulmonary conditions. In this review, we summarize the essential metabolic and signaling pathways driving the LLM formation in chronic obstructive pulmonary disease, pulmonary fibrosis, tuberculosis, and acute lung injury.

## 1. Introduction

Pulmonary diseases profoundly impact human health and productivity, with significant economic ramifications [1]. These conditions affect individuals across all demographics, from infants to the elderly [1,2,3]. One study reported that over 500 million people in the world were afflicted by chronic respiratory disease in 2017, nearly doubling since 1990 [4]. While pulmonary diseases have historically been prevalent in low- and middle-income countries, their incidence has been on the rise in wealthy countries like the UK and the US over the last decade [5,6]. For instance, in England, the healthcare cost related to chronic obstructive pulmonary disease (COPD) totaled GBP 1.50 billion in 2011, with projections estimating an additional GBP 1 billion by 2030 [7]. In the US, direct health costs for pulmonary diseases exceeded USD 32 billion in 2010 [8]. Acute respiratory diseases such as acute respiratory distress syndrome (ARDS) or pneumonia, frequently necessitate Intensive Care Unit (ICU) admissions [9,10,11], underscoring their significant burden on healthcare systems. Pulmonary diseases are associated with high morbidity, mortality, and unpredictable exacerbations requiring prompt intervention [12,13,14]. However, despite their impact, research and treatment for pulmonary conditions lag behind those for diseases like cancer or cardiovascular disorders. Closing this gap is crucial for improving outcomes and reducing the burden of pulmonary diseases worldwide.

The human lung is one of the vital organs for delivering oxygen to blood and cells, perpetually exposed to various foreign substances [15,16,17]. Bacteria or inhaled noxious gases, such as cigarette smoke (CS), can directly trigger an immune response and damage the lung tissue [18]. Since Metchnikoff’s elucidation of macrophages and phagocytosis, immunological research has been intimately tied to these versatile cells [19]. Different types of macrophages are present in different organs, boasting a plethora of surface receptors that confer them with diverse immune functions [19]. Pulmonary macrophages emerge as the key players in regulating acute or chronic inflammation in the lung constituting the predominant type of immune cells under homoeostatic conditions [17]. Pulmonary macrophages are tasked with manifold functions, such as maintenance of homeostasis, microbial clearance, responding to external stimuli, and degrading cell debris and excessive lipids [20,21]. Two primary types of pulmonary macrophages exist in the lung, named alveolar macrophages (AMs) identifiable by CD11c expression, and interstitial macrophages (IMs) characterized by CD11b expression [22,23]. AMs, residing in the alveolar space, play a central role in inflammatory lung diseases constituting over 90% of the immune cell population in the normal bronchoalveolar lavage (BAL) fluid [24].

AMs, surrounded by alveolar epithelial cells, are susceptible to lung-injury-induced epithelial damage, triggering pro-inflammatory transformation [25,26]. In the pro-inflammatory M1 state, they will release pro-inflammatory cytokines/chemokines, increase the secretion of oxygen metabolites, and exhibit enhanced phagocytic activity [27,28,29]. This transition to the M1 phenotype involves the participation of carbohydrate kinase-like protein (CARKL), a critical regulator of metabolic control in pro and anti-inflammatory immune responses [30]. The metabolic status and function of macrophages are intricately linked [31] with macrophage polarization influenced by the tricarboxylic acid cycle [32]. A recent study has revealed that *mammalian targets of rapamycin* (*mTOR*) deficiency in mice results in dysregulated fatty acid oxidation and amino acid pathways in AMs [33]. Functionally, amino acid and lipid metabolism may significantly support the normal development of AMs. Stimuli-induced macrophage activation prompts mTOR activity, enhancing sterol regulatory element-binding transcription factor (SREBP1a) function and lipid synthesis [34]. The augmented lipid synthesis supplies essential lipids for actin cytoskeleton and plasma membrane integrity, thereby bolstering phagocytosis [34]. In addition, lipid synthesis is also connected to the cytokine release by facilitating endoplasmic reticulum expansion [34,35].

Despite not being traditionally associated with lipid metabolism, the lung actively participates in pulmonary lipid transfer [36]. A key mediator of gas exchange in the lung is the pulmonary surfactant, a mixture vital for maintaining normal lung function [37]. Comprising primarily phospholipids (~90%) and surfactant-associated proteins (~10%), including surfactant proteins (SP) A, B, C, and D [37,38], the pulmonary surfactant plays a pivotal role in host defense, with SP-A and SP-D particularly studied in pulmonary research [39,40].

Various types of lung injuries can dysregulate pulmonary lipid metabolism, leading to the accumulation of damaged lipids [41,42]. AMs are conferred with phagocytic ability, which plays a crucial role in scavenging invaded foreign substances and is essential in regulating lipid catabolism [37,43]. Scavenger receptors (SRs) are one of the macrophage receptor families that is crucial in maintaining lipid metabolism [44]. SRs are similar to cluster of differentiation 36 (CD36) and lectin-like oxidized low-density-lipoprotein receptor-1 (LOX-1) that are known to participate in the lipid uptake [45,46,47]. During lung injuries, damaged or modified pulmonary lipids accumulate in AMs, potentially affecting macrophage apoptosis [48]. CD36, known for its role in binding to proteins that regulate oxidized low-density lipoprotein (oxLDL), contributes to excess storage of lipid droplets in macrophages [48,49,50], eventually leading to the formation of lipid-laden macrophages (LLMs).

Recent research has brought to light the potential significance of LLM formation across a broad spectrum of pulmonary diseases. Mounting evidence indicates that LLMs are not just a laboratory phenomenon but are indeed present in patients afflicted with diverse pulmonary conditions. In this review, we have compiled a comprehensive summary of the known inducers and essential genes involved in the formation of LLMs (Table 1). Specifically, we delve into the intricate mechanisms underlying LLM formation in COPD, pulmonary fibrosis (PF), tuberculosis (TB), and acute lung injury (ALI). By dissecting these pathways, we aim to elucidate the pivotal role of LLMs in the pathogenesis of these debilitating pulmonary diseases, shedding light on potential therapeutic targets and strategies.

## 2. Significance of LLMs in Lung Inflammation

LLMs, also known as foam cells or foamy macrophages, have been studied in the development of atherosclerosis [66,67], where they are considered one of the central mechanisms of atherosclerotic lesion formation and are known as the hallmark of the disease [68]. Cellular lipid metabolism is impaired with pathological injuries and results in the formation of LLMs [69]. Although the biogenesis of LLMs remains unclear, it is believed that LLMs play a crucial role in the pathogenesis of various diseases. For example, LLMs are associated with chronic inflammatory diseases like multiple sclerosis [70]. One study has demonstrated that the levels of both proinflammatory genes and cytokines were increased when oxLDL activated the CD36-Toll-like receptors 4 (TLR4)-Toll-like receptors 6 (TLR6) pathway in murine macrophages [71]. Furthermore, cholesterol crystal levels in macrophages are reported to be associated with defective autophagy and linked to proatherogenic inflammasome activation [72]. These findings suggested that excess uptake of lipids and the formation of LLMs could be deleterious to macrophage function and further promote inflammation.

Pro-inflammatory cytokines like TNF, IL-1α, and GM-CSF are reported to regulate lipid metabolism; meanwhile, TGF-β was reported as one of the central fibrotic markers actively participating in LLM formation [53,73,74]. The burden of macrophages in clearing excessive lipids significantly increases during the pro-inflammatory process and thus promoting the formation of LLMs [34,53,75]. Macrophage TGF-β plays a pivotal role in wound healing and promoting M2 macrophage activation; it is reported that inhibiting TGF-β-induced lipid droplets might change M2 macrophages back to their M1 phenotype [76,77,78]. The discussion regarding whether LLMs are more prone to develop from M1 or M2 macrophages is still undecided when studying pulmonary diseases. The activation of Toll-like receptor signaling by immune responses will encourage lipid accumulation and further enhance the LLM formation and inflammatory response [79]. This process may help attenuate the damage caused by infection, but it is anticipated to have detrimental effects on chronic metabolic or inflammatory disorders [67,79,80]. From this point, chronic mediators emerge, with pro-fibrotic mediators becoming prominent in the phenomenon of LLM formation. Therefore, it would be difficult to define LLMs for either M1 or M2 macrophages, considering their unique behavior. Instead, LLMs should be considered as a different stage located “outside” of pro-inflammatory or anti-inflammatory macrophages. The presence of LLMs has been reported in various pulmonary diseases, and their disappearance is also associated with the attenuation of injuries [28,53,58]. Thus, reducing the formation of LLMs could be a beneficial approach for pulmonary disease. Nevertheless, although strategies against LLMs in atherosclerosis have been investigated, studies in pulmonary diseases are limited [81,82]. Future studies, including clinical trials, are anticipated to discover and address these matters.

Studies have revealed a possible connection between cholesterol and lipoproteins in pulmonary lipid homeostasis [83]. LLMs were reported by healthcare specialists back in the late 90s as a potential biomarker for pulmonary and gastric aspiration [84,85]. The presence of LLMs was identified in multiple pulmonary diseases, such as COPD, idiopathic pulmonary fibrosis (IPF), asthma, and TB, suggesting their crucial role in the pathogenesis of pulmonary diseases [28,53,56,63]. Additionally, LLMs are observed in pulmonary alveolar proteinosis (PAP), a rare disorder characterized by the accumulation of surfactant and lipid droplets in AMs within the alveolar region, which mirrors the traditional pathway for LLM formation and underscores the potentially detrimental effects of LLMs [64,86]. Recent research has implicated oxidative stress as a potential regulator of PAP-induced LLM formation, with studies showing promising results using antioxidants like N-acetylcysteine (NAC) to alleviate PAP symptoms [65]. These findings shed light on potential therapeutic avenues for managing pulmonary diseases associated with LLM formation.

The high levels of lipids in the damaged lung can undergo oxidation by reactive oxygen species (ROS), a process known as lipid peroxidation [87]. ROS, including superoxide anions, are generated in various cellular processes, especially in lung cells [88]. Under normal conditions, oxidative homeostasis is maintained by abundant antioxidant capacity. However, exogenous oxidants can stimulate ROS production, leading to elevated oxidative stress in the lung and triggering various cellular responses [89,90]. The excess production of ROS leads to elevated oxidative stress in the lung triggering a range of diverse cellular responses. Pulmonary surfactants act as a barrier against inhaled noxious particles and pathogens, with pulmonary cells generating ROS to remove external substances [91]. On the other side, although ROS is essential for clearing the pathogen, it impairs the function of surfactants by oxidizing the phospholipids [92]. Evidence suggests that the rise of cellular oxidative stress is associated with inflammation and cellular apoptosis [93]. ROS serves as an important molecular mechanism regulating cell function and contributes to pulmonary diseases, including PF, ALI, TB, and COPD [89,94,95,96].

Research indicates that both passive and active cigarette smokers experience higher levels of oxidative stress, believed to be caused by the abundant presence of ROS in CS [89,90]. Consistently, clinical reports suggest that the level of hydrogen peroxide (H_2_O_2_) has a significant positive correlation with smokers and COPD patients compared to nonsmokers [97]. The inflammation-induced abnormal ROS level will lead to the recruitment of immune cells, including AMs, neutrophils, and T lymphocytes [98]. Under normal conditions, AMs could degrade and clear the damaged lipids in time to maintain pulmonary lipid hemostasis [99]. The rise of oxidative stress might oxidize the lipids and impair the phagocytic functions of AMs. In addition, high oxidative stress and oxidative lipids are well-known inducers of inflammation. Continuous exposure to oxidative environments and increased levels of inflammatory genes will affect granulocyte-macrophage colony-stimulating factor (GM-CSF), which is essential for degrading the uptake of lipids [100,101]. Dysregulation of GM-CSF leads to PAP, characterized by lipid accumulation [101]. Nevertheless, the potential mechanisms between pulmonary disease pathogenesis and LLMs have not been well studied. Here, we summarize the mechanisms and the role of LLM formation in pulmonary diseases, providing a comprehensive overview of their reported pathways, with a particular focus on pulmonary macrophages as the primary source of LLMs.

## 3. LLMs in COPD

COPD, a chronic respiratory condition characterized by airflow restriction, continues to be a leading cause of mortality worldwide [102]. The diagnostic criteria of COPD follow the Global Initiative for Chronic Obstructive Lung Disease (GOLD) guidelines, which classify the disease based on airflow limitation measured by spirometry, ranging from mild (GOLD I) to very severe (GOLD IV) [14]. The risk factors of COPD generally fall into two categories: genetic predisposition and environmental factors [14,103]. A prominent genetic factor linked to COPD is the deficiency of *α-1 antitrypsin*, a key inhibitor of elastase, which accounts for around 2% of COPD cases [104]. The major risk factor for COPD lies within the environmental factors, CS being the major environmental risk factor, inhalation of which could trigger an inflammatory response and oxidative stress in the lungs [28]. The phenotype of pulmonary LLMs was observed in the 1970s, but the mechanisms of LLM formation in the lungs remain understudied [105,106].

Morissette and colleagues have reported that CS-induced inflammation hampered lipid metabolism and facilitated lipid accumulation in murine pulmonary macrophages [107]. By exposing the *IL-1α*-deficient mice to CS, they discovered that lipid accumulation transformed BAL fluid to a cloudy form [107]. They also demonstrated that mice receiving GM-CSF inhibition exhibit comparable outcomes following CS exposure [107]. GM-CSF is crucial for differentiating the monocytes into AMs, and the *GM-CSF*-deficient mice also exhibit abnormal surfactant catabolysis in AMs [108]. A recent study has reported that the transcriptomic profile of AMs in COPD patients exhibited a significant change in lipid metabolism compared to healthy controls [102]. AMs are the most abundant cell type in the alveolar space in COPD and actively participate in pulmonary lipid metabolism by contributing approximately 50% surfactant degradation [109]. Interestingly, the study found that the CS-induced dysregulation of phospholipids, cholesteryl esters, and monoacylglycerols is associated with GOLD levels [102]. These reports point to AMs as the primary source of LLMs in the lungs.

Cholesterol esters are one of the primary components for lipid formation [110]. Studies have shown that acetyl-CoA acetyltransferase-1 (ACAT-1), primarily expressed in macrophages, has the ability to esterify cholesterol and thus participates in lipid droplet formation [111,112,113]. Scientists have observed excess lipid accumulation in lung macrophages from mice exposed to CS in an emphysema model [51,52,114,115]. These studies primarily investigated the dysfunction of SP-D, a critical factor in reducing oxidative and inflammatory responses in AMs [51,52]. The loss of SP-D has increased the lung oxidant levels, a contributor to lipid peroxidation, thereby elevating ROS levels. This cascade further accelerates the oxidation of cholesterol or phospholipids, consequently promoting the formation of oxLDL. In line with this observation, we found that CS-induced LLMs have significantly higher ROS levels compared to untreated AMs, suggesting that oxidative stress plays a critical role in LLM formation [28]. The study from our group has shown that overexpressing the myeloid origin *miRNA-103a* (*miR-103a*) could inhibit the LLMs induced by CSE via suppressing LDLR [28]. Moreover, the overexpressed *miR-103a* can simultaneously decrease the ROS level accompanied by the CSE treatment [28]. This observation can also be supported by the research from Hsieh et al., by which treating SP-D could help to prevent the formation of oxLDL-induced LLMs and oxidative-stress-induced emphysematous change [52]. SP-D was reported to have a protective role in COPD by reducing the oxidative stress in the alveolar area, and it is expected that increasing the SP-D level could benefit the injured lung [116]. Hirama and colleagues have further confirmed that increasing SP-D could reduce the lipid accumulation in macrophages in mice exposed to CS for 6 months [51]. Similar findings were reported by Poliska et al., where LDLR expression in AMs from COPD patients was higher than the controls [117]. These changes collectively suggest that CS exposure disrupts lipid homeostasis, leading to the oxidation of lipids into oxLDL, which is subsequently taken up by the AMs to form LLMs.

## 4. LLMs in PF

PF is a heterogeneous, progressive, and chronic inflammatory lung disorder, leading to the scarring and stiffening of lung tissue, ultimately causing respiratory failure [118,119]. PF has emerged as a significant global pulmonary health concern, impacting over 5 million people worldwide, with a median survival of 3–5 years post-diagnosis [118,119,120]. PF is characterized by fibrotic progression resulting from repetitive scarring of the lungs and excessive deposition of collagen, which can disrupt the normal alveolar architecture [121,122,123,124]. However, the etiology of metabolic mechanisms, such as the formation of LLMs in PF, remains incompletely understood. Like COPD, the inhalation of noxious particles such as CS, dust, and silica is considered a critical environmental factor contributing to PF pathogenesis [125,126,127]. As the first line of defense of the pulmonary immune system, AMs are actively enrolled in maintaining lipid homeostasis. While in contact with the invaded particles, AMs are polarized into either the “classically activated” M1 macrophages that produce inflammatory cytokines or “alternatively activated” anti-inflammatory M2 macrophages which are commonly considered to participate in the pathogenesis of fibrosis [128,129,130].

Yasuda et al. have reported that the phenotype of LLMs was observed in a rabbit model [131]. An increase in phospholipid level and the recruitment of BAL cells were found together after treatment with bleomycin [131]. The elevation of phospholipid levels in BAL cells is undoubtedly a clear indicator of LLM formation, particularly considering that AMs are the predominant cell type in BAL fluid [132]. Interestingly, although the emergence of LLMs is typically associated with the advanced stages of fibrotic lung disease, LLM formation also might signify the dysregulation of lipid metabolism in AMs, potentially occurring earlier in the disease progression. In support of this, Romero et al. observed LLM formation on day 3 post bleomycin administration in mice [53]. This observation aligns with the time when cholesterol and triglycerides significantly increased [53]. However, the trichrome staining indicates that lung collagen appears to be substantially increased on day 7 after bleomycin administration, suggesting the presence of fibrosis. Romero et al. further showed that pretreating mice with GM-CSF could decrease the quantity number of LLMs and thus attenuate bleomycin-induced PF by suppressing lipogenic-related genes such as *fatty acid synthase* (*FASN*) [53]. Furthermore, Romero et al. indicated that deleting the *ATP binding cassette subfamily G member 1* (*ABCG1*) gene in mice increases the number and size of LLMs post-bleomycin administration [53]. The authors observed that treatment with bleomycin significantly increased transforming growth factor-beta (TGF-β1) secretion by M2 macrophages, which is a well-known inducer of fibrosis, suggesting the possible connection between LLMs and fibrosis progression [53,133]. Besides the bleomycin model, another study has demonstrated that the expression of lipid efflux transporters *ATP-binding cassette transporter A1* (*ABCA1*) and *ABCG1* decreased in a nitrogen-mustard-treated mouse model [54]. Meanwhile, lipid-handling receptors like *farnesoid-X receptor* (*FXR*) and *CD36* have increased, suggesting more phospholipids and cholesterol were taken up by macrophages [54]. With regard to SRs, Kwak and colleagues have found that fewer oxidized phospholipids (oxPL) induced scarring and the formation of LLMs in *CD36*-null mice compared to wild-type (WT) mice after treating oxPL-derivative oxidized phosphocholine [55].

## 5. LLMs in TB

The literature suggests that nearly one-third of the global population is a potential carrier of *Mycobacterium tuberculosis* (Mtb), the bacterium that causes TB [134,135,136]. TB, a chronic inflammatory disease characterized by the development of granulomas, causes approximately 1 million deaths worldwide [137]. Typically, inhaled pathogen like Mtb will be phagocytosed and degraded by Ams; however, Mtb evades this process by adapting to ROS or compromising phagosome function [96]. Mtb remaining in the AMs causes a series of changes, starting from the alternation to cellular metabolism, which directly induces LLM formation [138,139]. LLMs would further enhance the inflammatory response by recruiting immune cells to build up granulomas [139]. Therefore, comprehending the physiological and pathological roles of AMs and LLMs is important in TB research. The accumulation of lipids, containing cholesteryl esters or triglycerides, sets the stage for LLM formation. Studies have indicated that inhibiting tumor necrosis factor receptor (TNFR) signaling via the activation of the mammalian target of rapamycin complex 1 (mTORC1) could reduce triglycerides secretion, thus rescuing altered hepatosteatosis [73,140].

Recent reports of liver X receptors (LXRs) on macrophages have demonstrated their function in mediating macrophage lipid transport [141,142]. The activation of LXRs has been reported to participate in cholesterol metabolism by mediating lipid-related genes such as *ABCA1*, *ABCG1*, and *apolipoprotein E* (*apoE*) [143,144]. The study by Korf et al. demonstrated that the number of LLMs has increased in Mtb-infected *LXRα*^−/−^ or *LXRα*^−/−^*LXRβ*^−/−^ mice, suggesting that LXR could be a regulator of LLMs [56]. Another cholesterol mediator, cholesterol 25-hydroxylase (CH25H), was found to be upregulated during granuloma development [57]. A reduction in the number of LLMs was observed in the Raw 264.7 cells after CH25H silencing, followed by a decrease in adipocyte differentiation-related protein (ADRP) [57]. Furthermore, Jaisinghani et al. published one study showing that the knockdown of *diacylglycerol acyltransferases-1* (*DGAT1*) could inhibit LLM formation [58]. DGAT1 plays a pivotal role in regulating triglyceride levels, resulting in a reduction in the number of lipid droplets when DGAT1 is suppressed. Additionally, macrophage receptors also play a critical role due to their phagocytic behavior. Furthermore, one study has noted that LLM formation is suppressed in *testicular orphan nuclear receptor 4* (*TR4*) deficiency mice by inhibiting CD36 expression [59]. Similarly, the knockdown of *peroxisome proliferator-activated receptor γ* (*PPARγ*) could inhibit the dysregulated lipid biogenesis, consequently inhibiting LLM formation in THP-1 cells [60]. Functionally, Mtb infection induces the expression of *PPARγ*, thereby further increasing CD36 levels to facilitate the uptake of oxLDL, promoting the formation of LLMs. On the other hand, another study has shown that Mtb-induced LLM formation could be inhibited by the IL-4 and signal transducer and activator of the transcription 6 (STAT6)-driven pathway [61].

## 6. LLMs in ALI and Pneumonia

ALI is characterized by a rapid onset of respiratory failure, significant hypoxemia, and the presence of bilateral infiltrates on the chest radiograph, which indicates pulmonary edema [145,146,147]. The common direct cause of ALI includes inhalation of noxious gas, gastric aspiration, and pneumonia [146]. In the *Klebsiella pneumoniae*-induced experimental ALI model, both the number of LLMs and the inflammatory markers were increased simultaneously, indicating a connection between the immune response and the LLM formation [148]. The authors demonstrated that the formation of LLMs may result from impaired SR function. Additionally, in the severe acute respiratory syndrome (SARS) mouse model, LLMs were observed following infection induced by murine hepatitis virus strain 1 (MHV-1) [62]. Functionally, *inflammatory mediator long pentraxin 3* (*PTX3*)-deficient mice exhibited more severe exacerbated injury conditions compared to the WT control [62]. In addition, LLMs were reported to be a biomarker for aspiration-induced pneumonia in the late 90s [84,149]. Most recently, researchers reported that LLMs are also associated with vaping-associated lung injury [150,151]. Beginning with the proliferation of the electronic cigarette (E-cigarette) industry, LLMs were also observed and reported to serve as a potential biomarker in E-cigarette or vaping product use-associated lung injury (EVALI) in youth [151,152]. Future investigations may offer enhanced insights into the contribution of LLMs to the development of EVALI.

## 7. LLMs in Lung Cancer

Statistics from the American Cancer Society in the year 2023 show that lung and bronchus cancer was ranked second place in the estimated new cases and ranked first in the estimated deaths in both males and females [153]. More than 80% of lung cases are non-small cell lung cancer (NSCLC), which can be further classified as adenocarcinoma, large cell carcinoma, and squamous cell carcinoma [154]. Wang et al. have reported that the evaluated level of nitric oxide synthase 2 (NOS2) is closely associated with the presence of LLMs [155]. The authors find that deleting NOS2 has significantly reduced the number of LLMs and the dysregulation of the lipid metabolism in L-IkkαKA/KA mice [155]. Another observation based on the lung squamous cell carcinoma murine model from Dai and colleagues demonstrated that the ablation of *LXRα* and *LXRβ* promotes the accumulation of LLMs [156]. Moreover, LLMs were also observed in pulmonary sclerosing pneumocytoma (PSP) [157]. Fan and colleagues have reported that LLMs or foam-like macrophages were observed in a 31-year-old PSP patient [158]. Nevertheless, there are also studies showing that LLMs or foam cells could be observed while applying cancer therapy [159,160]. Macrophages have been reported as an important candidate for both diagnoses and serving as potential therapeutic targets for cancers [161]. However, further research is needed to understand the underlying mechanisms involved.

## 8. Targeting LLMs for the Treatment of Treat Lung Diseases

Though macrophages are considered potential diagnostic tools for pulmonary disease, their clinical relevance may vary among individuals [162,163]. Nevertheless, as the “gatekeepers” of the respiratory system, the mechanisms of macrophages cannot be overlooked. By releasing proinflammatory cytokines, macrophages could recruit monocytes into the lung and become AM-liked cells [164,165]. As discussed before, inflammation is closely related to the formation of LLM. The formation of LLM, from most of the studies mentioned above, is considered to be harmful for most disease conditions. The immunometabolism of macrophages is compromised when they uptake excessive lipid droplets and turn into LLMs [34,166]. Therefore, it is crucial to develop methods to counteract this phenomenon. The process of LLM formation is closely related to lipid transportation in the alveoli areas, which could be interfered with by different types of injuries [167]. Although various potential pathways have been reported to illustrate the mechanism of LLM, it remains largely unknown. Therefore, therapeutic options against LLM formation in pulmonary diseases are limited and it is worth looking deeper into its mechanism. One of the essential processes to consider during the formation of LLMs is the dysregulated lipid metabolism from the lung injury [168]. The injured lung tends to increase the number of damaged lipids, which is favored and easily oxidized by the ROS generated from the inflammatory response, while lung macrophages react to different stimuli [90]. ROS is the fundamental mechanism of macrophages, allowing them to remove the phagocytosed microorganisms [27]. In addition, oxidative stress is widely observed in various pulmonary diseases, and it is closely associated with disease pathology [94,169,170,171]. To counter the oxidative stress, scientists applied antioxidant therapy to inhibit LLM. Ghodsian et al. showed that using the antioxidant 7,8-dihydroneopterin effectively reduces the formation of LLMs [172]. Functionally, they observed that 7,8-dihydroneopterin decreased the expression of CD36 in U937 cells [172]. Another study from Sung et al. has revealed that NAC could help to decrease the ROS level and LLM formation by down-regulating CD36 expression [173]. SRs play a central role in LLM formation, receptors like CD36 that uptake lipids could be regulated by the elevated level of ROS accompanied by the appearance of lung injury [174]. To support this view, one study has reported that dipeptidyl peptidase-4 inhibitor Linagliptin could mitigate the number of LLMs by decreasing the expression of CD36 and lipoprotein receptor-1 (LOX-1) [175]. Another study by Chen and colleagues has shown that Epigallocatechin-3-gallate has attenuated the formation of LLMs in THP-1 cells by decreasing the expression of scavenger receptor A [176]. The appearance of oxLDL is also a clear indication of the increased oxidative stress, which could result in lipid peroxidation and further increase the portion of oxidized lipids [28,87]. Song et al. have reported that the Food and Drug Administration (FDA)-approved Zafirlukast, a drug used to treat asthma, could prevent macrophages from turning into LLMs [177,178]. They have shown that the use of Zafirlukast successfully down-regulated the expression of CD36 and LOX-1 [178]. Hoeffner et al. have found that another FDA-approved drug, Verteporfin, effectively reduced the accumulation of lipids to decrease the process of LLM formation [179]. The ox-LDL-induced lipid accumulation was also reported to be reduced by using aspirin eugenol ester, by Liu and colleagues [180]. Yatera et al. reported the presence of club cells could mediate the expression of LOX-1, CD36, and matrix metalloproteinases (MMPs) −2, −9 and −12 [181]. The club cell-ablated mice have more LLMs compared with the WT mice after inhaling crystalline silica [181]. Although the above treatments have been shown to block LLM formation, further studies are needed to assess the potential therapeutic effects against lung diseases.

## 9. Conclusions and Future Perspectives

**Figure 1:**
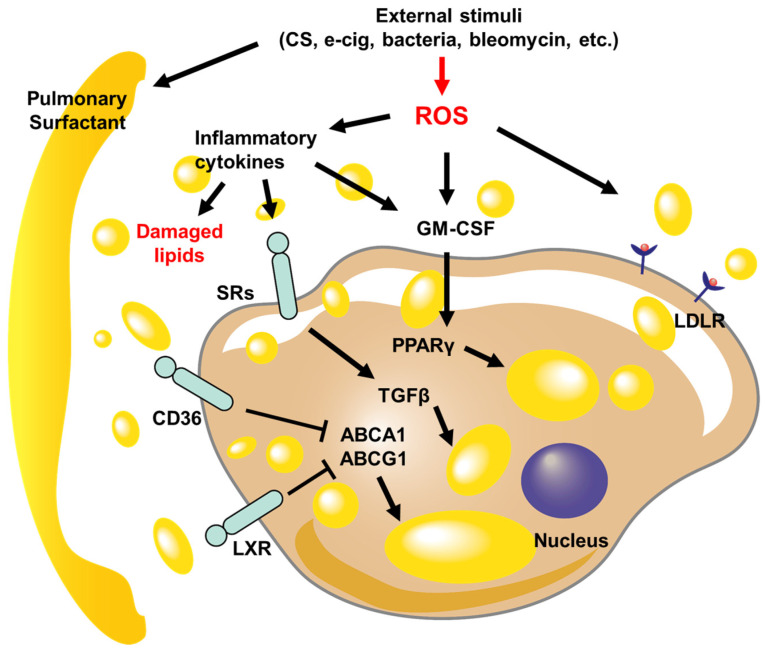
Schematic diagrams of signaling pathways regulate LLM formation in pulmonary diseases. The lung injury caused by external stimuli disrupts lipid metabolism, leading to damage to the pulmonary surfactant and an increase in reactive oxygen species (ROS) levels. The inflammatory cytokines that are released increase the expression of scavenger receptors (SRs), cluster of differentiation 36 (CD36), liver X receptors (LXRs), and low-density lipoprotein receptor (LDLR), which enables the transport of damaged or oxidized lipid droplets within macrophages. As the lipid levels within the cell rise, the activity of ATP-binding cassette transporter A1 (ABCA1) and ATP-binding cassette subfamily G member 1 (ABCG1) is suppressed, while transforming growth factor-beta (TGFβ) and peroxisome proliferator-activated receptor γ (PPARγ) further contribute to the accumulation of lipids.

In conclusion, this review comprehensively examines the formation of LLMs in various pulmonary diseases, providing insights into their pathophysiological significance and therapeutic implications. Through meticulous examination of the literature, we have synthesized a wealth of knowledge regarding the formation, regulation, and functional consequences of LLMs in diverse pulmonary conditions. By consolidating the key genes and signaling pathways contributing to LLM formation as depicted in Figure 1, we have illuminated the complex mechanisms underlying LLM formation. Furthermore, our review delineates the intricate signaling pathways and molecular mechanisms that govern LLM formation and function. From dysregulated lipid metabolism and oxidative stress to cytokine-driven immune responses and macrophage polarization, a complex network of interactions orchestrates the dynamics of LLMs in pulmonary diseases. Understanding these pathways not only enhances our comprehension of disease pathophysiology but also unveils novel therapeutic targets for intervention.

In light of these findings, we propose a paradigm shift in the management of pulmonary diseases, wherein LLM-targeted therapies hold promise for improving clinical outcomes. By modulating LLM formation, function, or clearance, therapeutic interventions have the potential to attenuate inflammation, promote tissue repair, and restore pulmonary homeostasis. From pharmacological agents targeting scavenger receptors and lipid metabolism to immunomodulatory strategies aimed at modulating macrophage phenotypes, a diverse array of therapeutic modalities is being explored to harness the therapeutic potential of LLMs. However, while the prospects are promising, several challenges and unanswered questions remain. Further elucidation of the molecular mechanisms driving LLM formation, the development of robust preclinical models, and rigorous clinical validation of therapeutic interventions are needed to translate these findings into clinical practice. Additionally, the heterogeneity of LLM populations and their context-dependent functions warrant further investigation to tailor therapeutic strategies to specific disease contexts and patient populations.

In summary, this review underscores the pivotal role of LLMs in pulmonary diseases and highlights their potential as therapeutic targets. By unraveling the complexities of LLM biology and translating these insights into clinical practice, we can usher in a new era of precision medicine for the management of pulmonary diseases, ultimately improving patient outcomes and quality of life.

## Figures and Tables

**Table 1:** Overview of inducers and genes in LLM formation in pulmonary diseases.

Disease	Inducer	Genes Involved in LLM Formation	Reference
COPD	cigarette smoke	*SP-D*, *LDLR*, *microRNA-103a*	[28,51,52]
PF	bleomycin	*ABCG1*, *TGF-β1*	[53]
	nitrogen mustard	*ABCA1*, *FXR*, *CD36*	[54]
	oxPL	*CD36*	[55]
TB	*M. tuberculosis*	*LXRs*	[56]
		*CH25H*, *ADRP*	[57]
		*DGAT1*	[58]
		*TR4*, *CD36*	[59]
		*PPAR* *γ*	[60]
		*STAT6*	[61]
ALI	MHV-1	*PTX3*	[62]
Asthma	-	-	[63]
PAP	-	-	[64,65]

COPD: chronic obstructive pulmonary disease; PF: pulmonary fibrosis; TB: tuberculosis; ALI: acute lung injury; PAP: pulmonary alveolar proteinosis; MHV-1: murine hepatitis virus strain 1; SP-D: surfactant proteins D; LDLR: low-density lipoprotein receptor; ABCG1: ATP binding cassette subfamily G member 1; TGF-β1: transforming growth factor-beta1; ABCA1: ATP-binding cassette transporter A1; oxPL: oxidized phospholipids; FXR: farnesoid-X receptor; LXRs: liver X receptors; CH25H: cholesterol 25-hydroxylase; ADRP: adipocyte differentiation-related protein; DGAT1: diacylglycerol acyltransferases-1; TR4: testicular orphan nuclear receptor 4; PPARγ: peroxisome proliferator-activated receptor γ; STAT6: signal transducer and activator of the transcription 6; PTX3: long pentraxin 3.

## Data Availability

Not applicable.
